# Chemical chaperone, TUDCA unlike PBA, mitigates protein aggregation efficiently and resists ER and non-ER stress induced HepG2 cell death

**DOI:** 10.1038/s41598-017-03940-1

**Published:** 2017-06-19

**Authors:** Jagadeesh Kumar Uppala, Amina R. Gani, Kolluru V. A. Ramaiah

**Affiliations:** Department of Biochemistry, University of Hyderabad, Hyderabad, 500046 Telangana India

## Abstract

Stress induced BSA (bovine serum albumin) protein aggregation is effectively mitigated *in vitro* by TUDCA (tauroursodeoxycholic acid) than by PBA (4- phenylbutyric acid), chemical chaperones approved by FDA for the treatment of biliary cirrhosis and urea cycle disorders respectively. TUDCA, unlike PBA, enhances trypsin mediated digestion of BSA. TUDCA activates PERK, an ER-resident kinase that phosphorylates the alpha-subunit of eukaryotic initiation factor2 (eIF2α) and promotes the expression of activated transcription factor 4 (ATF4) in HepG2 cells. In contrast, PBA induced eIF2α phosphorylation is not mediated by PERK activation and results in low ATF4 expression. Neither chaperones promote expression of BiP, an ER chaperone, and CHOP (C/EBP homologous protein), downstream target of eIF2α-ATF4 pathway. Both chaperones mitigate tunicamycin induced PERK-eIF2α-ATF4-CHOP arm of UPR and expression of BiP. TUDCA, unlike PBA does not decrease cell viability and it also mitigates tunicamycin, UV-irradiation and PBA induced PARP (poly ADP-ribose polymerase) cleavage and cell death. These findings therefore suggest that TUDCA’s antiapoptotic activity to protect HepG2 cells and PBA’s activity that limits tumor cell progression may be important while considering their therapeutic potential.

## Introduction

Protein homeostasis is critical to maintain cellular homeostasis, health and disease^1^. During or following their synthesis, the newly made polypeptides undergo necessary posttranslational modifications and are then folded correctly for all necessary intra- and inter-protein or molecular interactions and to carry out their functions. Maintenance of cellular proteostasis requires the cooperation and coordination of several pathways that include protein folding, degradation and trafficking. Depending on the nature of stress, unfolded proteins can accumulate in any of the cellular organelles like cytosol, endoplasmic reticulum (ER), mitochondria etc^2–4^. ER, a specialized subcellular organelle, is involved in the synthesis of lipids and secretory proteins, maintains an oxidative environment to ensure the formation of disulphide bonds in secretory proteins and is also a store for calcium which is required for the functioning of many of its chaperones. It regulates the synthesis and modification, folding, transport and degradation of secretory proteins. Disturbances in the ER homeostasis due to excessive protein synthesis beyond the capacity of protein folding, defective covalent modifications and protein degradation, changes in intracellular calcium levels, and oxidative stress leads to accumulation of misfolded or unfolded proteins in the lumen of the ER. Stressed ER activates unfolded protein response (UPR)^5–9^, an adaptive signaling pathway that is evoked to restore protein folding. UPR plays a role in cell survival and proliferation during metastasis^10, 11^, and declines during chronological ageing^12^. Chronic ER stress is a cause for diabetes, obesity, neurological disorders and cancer^10, 12–14^.

ER membrane consists of three ER stress sensors that sense the accumulation of unfolded proteins in ER lumen. These are: PERK (ER- resident eIF2α kinase), IRE1 (Inositol requiring enzyme), and ATF6 (Activated transcription factor 6). These are inactive in normal conditions and are bound by BiP/GRP78, an ER chaperone and master regulator of UPR. Release of BiP from these stress sensors during ER stress, results in their activation and leads to changes in translation and transcription. Activated PERK phosphorylates ser^51^ residue in the alpha-subunit of heterotrimeric eukaryotic initiation factor 2 (eIF2α) that is involved in the initiation step of protein biosynthesis. Phosphorylation of eIF2α occurs by various ser/ thr kinases such as heme-regulated inhibitor (HRI), double stranded RNA-dependent protein kinase (PKR), general control nonderepressible kinase (GCN2), and PKR-like endoplasmic reticulum kinase (PERK) that are activated in response to diverse stressors such as heme-deficiency or denatured proteins, viral infection, nutrient limitation or amino acid starvation and unfolded proteins respectively^16, 17^. Phospho-eIF2α, a stress, survival and suicidal signal^18^, sequesters and inhibits a rate limiting protein called eIF2B, a GDP/GTP exchange factor that recycles inactive eIF2.GDP to active eIF2.GTP^19, 20^ and thereby attenuates translational initiation of general mRNAs^17^.

Phosphorylated eIF2α, an integrated stress response, is also a signal for preferential translation of certain genes that code for transcriptional factors like ATF4, GCN4 and CHOP containing small upstream open reading frames (uORFs) which in turn induce genes involved in the synthesis of redox metabolism, amino acid metabolism, autophagy or cell death respectively^17, 21, 22^. A decline in general translation can also facilitate translation of some rare mRNAs due to decreased competition. Resumption in translation mediated by dephosphorylation of eIF2α through CHOP induced expression of GADD34, a cofactor of protein phosphatase-1 can occur during periods of adaptation^23–25^. However, if dephosphorylation of eIF2α happens before ER stress is relieved, it will cause additional burden on protein folding. Activation of IRE-1, the second arm of UPR in ER stressed conditions processes XBP-1 mRNA to a spliced form of XBP-1 that encodes genes for protein folding and degradation. Activated IRE-1-TRAF2 interaction leads to JNK activation or phosphorylation which in turn evokes signaling pathways involved in cell death or autophagy^26–28^. ER stress translocates protein ATF6, the third arm of UPR, to golgi, where it is processed by S1 and S2 proteases and becomes active that works together or separately with XBP1 to regulate UPR induced gene expression^9^.

Recent studies have identified several small molecules, compounds or drugs based on their ability to influence various components of UPR. These include molecules that (a) interact directly with some of the components of UPR signaling pathway; (b) reduce ER stress like chemical chaperones such as PBA, TUDCA and TMAO; (c) inhibit protein degradation; (d) carry antioxidant activity and (e) affect ER calcium signaling^29, 30^. These compounds are emerging as novel therapeutic targets to treat ER stress associated pathologies. Among these various compounds, chemical chaperones such as PBA, a low molecular weight chemical chaperone with a terminal aromatic substituted fatty acid, and TUDCA, a hydrophilic bile salt, are approved by US FDA to treat diseases such as urea cycle disorders and biliary cirrhosis respectively^31–33^.

While TUDCA is reported to have an antioxidant and antiapoptotic activity^34^, PBA is known as an ammonia scavenger in children with urea cycle disorders, attenuates ER stress and also inhibits histone deacetylase mildly^35^. Recent studies demonstrating TUDCA’s antiapoptotic role in many of the neurodegenerative diseases, retinal and, metabolic disorders like diabetes and obesity suggest a therapeutic role for TUDCA in many of these diseases^35–37^. Similarly PBA is also shown to have potential therapeutic effects to treat various diseases such as streptozotocin-induced diabetic nephropathy, hyperglycemic induced ER stress, cystic fibrosis, parkinson’s disease, ischemic injury, fibrosis, platelet aggregation, and hemoglobin disorders^38^. Recent studies also suggest that PBA acts as an anticancer agent by inhibiting growth and induce apoptosis in many cancers suggesting that its effect could vary depending on the cell type^34, 39–44^.

In a recent study, we reported that TUDCA mitigates heat or heat and DTT induced BSA protein aggregation *in vitro* and activates PERK in a concentration dependent manner in HepG2 cells^45^ suggesting that TUDCA promotes ER stress and activates UPR. This is in contrast to the reports by others, as mentioned above, that TUDCA reduces ER stress induced activation of UPR. In continuation of our previous work, we have studied here the importance of TUDCA and PBA on PERK activation, for their ability to prevent stress induced protein aggregation *in vitro*, ER stress induced UPR and cell death. Our observations suggest that TUDCA, but not PBA effectively mitigates stress induced BSA protein aggregation and enhances trypsin mediated BSA digestion *in vitro*. Unlike PBA which reduces cell viability, TUDCA is cytoprotective and promotes mild UPR. Both chaperones mitigate activation of PERK-eIF2α-ATF4 arm of ER stress induced UPR. However TUDCA suppresses tunicamycin, UV-irradiation and PBA induced PARP cleavage and HepG2 cell death suggesting that TUDCA’s antiapoptotic activity is not limited to ER stress induced cell death.

## Results

### PBA is not as efficient as TUDCA in relieving protein aggregation *in vitro*

**Figure 1:**
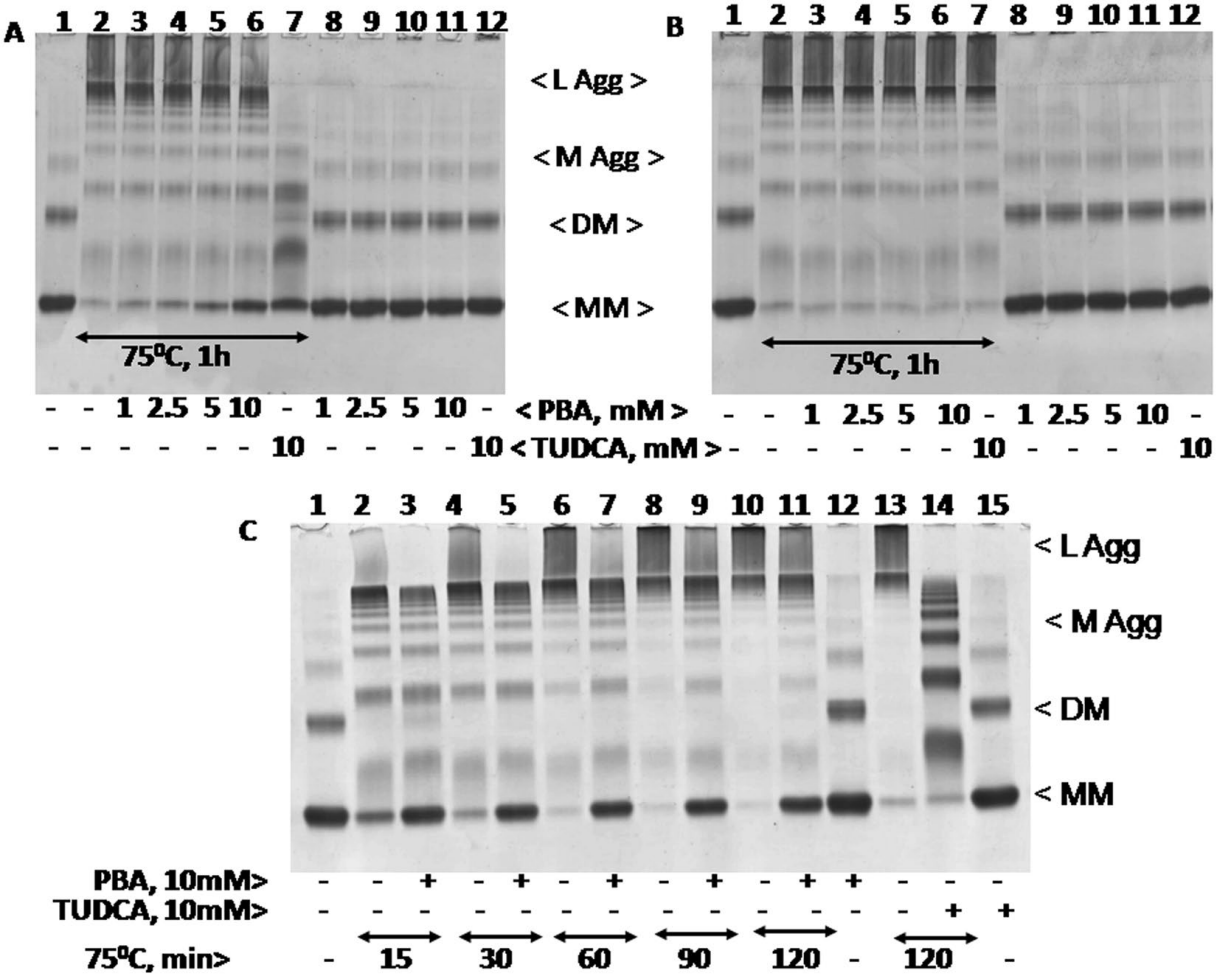
Heat induced aggregation of BSA in the presence of PBA or TUDCA: BSA (2 mg/ml) was heat treated at 75° C for 1 h in the presence and absence of different concentrations of PBA (1–10 mM) or 10 mM TUDCA either during heat treatment (Panel A) or after heat treatment (Panel B). Samples were analyzed by 8% Native-PAGE. The various lanes in Panel A and B are: 1, Control BSA; 2, heat treated BSA at 75° C for 1 h; 3–6, heat treated BSA in the presence of 1, 2.5, 5, and 10 mM PBA respectively; 7, heat treated BSA in the presence of 10 mM TUDCA; 8–11, native or control BSA in the presence of 1, 2.5, 5, and 10 mM PBA; 12, native BSA with 10 mM TUDCA. Abbreviations are as follows: L Agg and M Agg refer to large and medium aggregates; DM and MM refer to dimers and monomers respectively. Panel C represents heat induced aggregation of BSA at 75° C in the presence and absence of 10 mM PBA for different time periods (15, 30, 60, 90, and 120 min) or 10 mM TUDCA for 120 min. The various lanes are: 1, native BSA; 2, 4, 6, 8, and 10, BSA samples heat treated for 15, 30, 60, 90, and 120 min respectively; 3, 5, 7, 9, and 11, BSA samples heat treated for 15, 30, 60, 90, and 120 min respectively in the presence of 5 mM PBA; 12, native BSA with 5 mM PBA; 13, BSA heat treated for 120 min; 14, BSA heat treated for 120 min in the presence of 10 mM TUDCA; 15, native BSA with 10 mM TUDCA.

Previously, we observed TUDCA reduces heat, DTT, and also heat and DTT induced BSA aggregation *in vitro* ^45^. We have studied here the ability of PBA to relieve aggregation of BSA in the above mentioned conditions and compared to TUDCA. Heat treated BSA (75° C/1 h), was found mostly at the top of the 8% Native-PAGE (Fig. 1A, lane, 2 vs 1) suggesting that it was predominantly aggregated. As BSA dimers (DM) were reduced more significantly during heating, it is likely that heat induced formation of large aggregates are derived from dimers. Inclusion of PBA prevented heat induced BSA aggregation partly and protected BSA monomers (MM) in a concentration dependent manner (Fig. 1A, lanes, 3–6 vs 2). Analysis of the formation of heat induced large aggregates of BSA in the presence of TUDCA and PBA suggest that TUDCA prevents heat induced protein aggregation much more efficiently than PBA (Fig. 1A, lane, 7 vs 6). However, heat induced aggregation of BSA was relieved by TUDCA or PBA only when these agents were present during heating but not after heating (Fig. 1A vs B). In the absence of heating, BSA migration was unaffected by different concentrations of PBA or TUDCA (10 mM) compared to its control (lanes 8–12 vs 1).

Time course analysis of BSA aggregation in response to heat in the presence and absence of PBA or TUDCA was shown in Fig.1C. Large aggregates of BSA formed in response to heat treatment at 75° C were reduced and BSA monomers were protected significantly in the presence of 10 mM PBA (Fig. 1C, lane, 2–11). PBA however could not relieve the larger aggregates as efficiently as TUDCA (Fig. 1C, lane 14 vs 11). This may be due to the detergent like action associated with TUDCA that solubilizes higher aggregates. In the absence of heating, PBA and TUDCA did not affect BSA migration compared to its control (lane 12 and 15 vs lane 1). Previously, we showed that TUDCA and DTT reduced ANS mediated BSA fluorescence^45^. PBA also reduced here ANS mediated BSA fluorescence in a concentration dependent manner and more efficiently than TUDCA and DTT (see Supplementary Fig. S1A and B). PBA also reduced ANS-mediated BSA fluorescence in the presence of DTT more efficiently than TUDCA (see Supplementary Fig. S1C). These findings therefore suggest that PBA binds perhaps more strongly to the hydrophobic regions in BSA than TUDCA or DTT.

**Figure 2:**
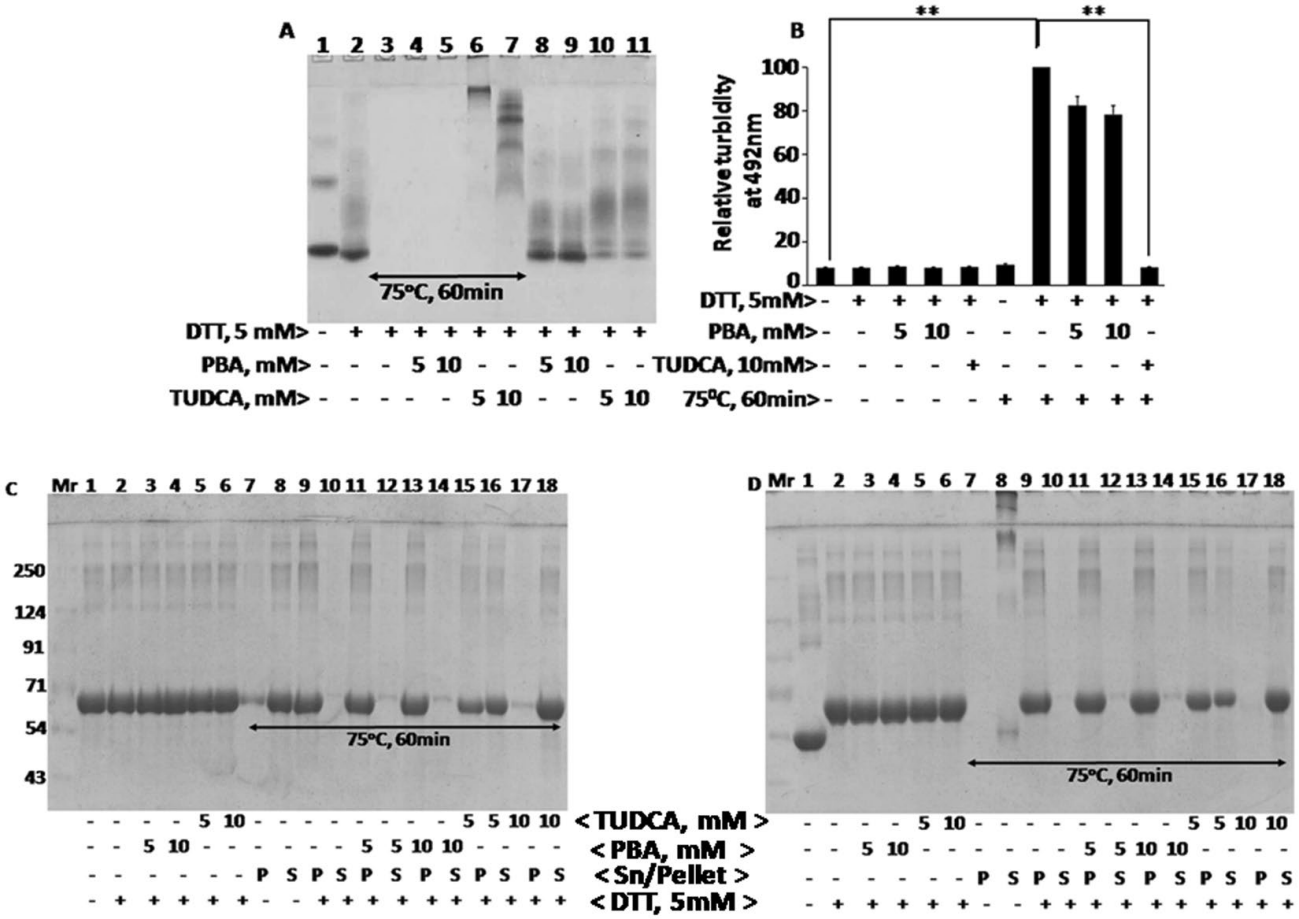
Heat and DTT induced BSA aggregation in the presence of PBA or TUDCA: Panel A BSA samples were treated with or without heat at 75° C for 1 h in the presence and absence of 5 mM DTT and PBA or TUDCA (5 and 10 mM). BSA aggregation was analyzed by 8% Native-PAGE. Various lanes are as follows: 1, BSA; 2, BSA + DTT; 3, BSA + DTT + heat; 4, BSA + DTT + 5 mM PBA + heat; 5, BSA + DTT + 10 mM PBA + heat; 6, BSA + DTT + 5 mM TUDCA + heat; 7, BSA + DTT + 10 mM TUDCA + heat; 8, BSA + DTT + 5 mM PBA; 9, BSA + DTT + 10 mM PBA; 10, BSA + DTT + 5 mM TUDCA; 11, BSA + DTT + 10 mM TUDCA. Panel B represents turbidity assay to determine heat and DTT induced BSA aggregation in the presence and absence of different concentrations of PBA or TUDCA. Turbidity was measured at 492 nm by spectrophotometer. Panel C and D represents heat (75° C for 60 min) and 5 mM DTT induced aggregation of BSA in the presence and absence of PBA or TUDCA (5 and 10 mM). After treatments, BSA samples were spun at 10, 000 RPM for 10 minutes and the pellet (P) and supernatant (S) fractions were analyzed by 12% reducing (Panel C) or non-reducing SDS-PAGE (Panel D). Various lanes are: 1, BSA; 2, BSA + DTT; 3, BSA + DTT + 5 mM PBA; 4, BSA + DTT + 10 mM PBA; 5, BSA + DTT + 5 mM TUDCA; 6, BSA + DTT + 10 mM TUDCA; lanes 7, 9, 11, 13, 15, and 17 are pellet fractions of BSA + heat, BSA + DTT + heat, BSA + DTT + 5 mM PBA + heat, BSA + DTT + 10 mM PBA + heat, BSA + DTT + 5 mM TUDCA + heat, and BSA + DTT + 10 mM TUDCA + heat respectively; and lanes 8, 10, 12, 14, and 16 are supernatant fractions. Data shown in Panel B are mean ± SD, (n = 3). The asterisks represent statistically significant differences (*P < 0.05, **P < 0.01). P value is calculated by t-test.

Higher concentrations of DTT or heat and DTT also promoted aggregates of BSA (Fig. 2). 5 mM DTT alone destabilized BSA, resulted in the loss of BSA dimers and monomers significantly, and lead to the formation of smaller aggregates (Fig. 2A lane 2 vs 1). Both TUDCA and PBA prevented DTT induced formation of smaller aggregates (lanes 8–12 vs 2). However, BSA protein disappeared altogether and could not enter into the native gel when it was treated with DTT and heat (75° C/1 h) (lane 3 vs 2 or 1) suggesting that heat coupled with DTT treatment promotes more complex and larger aggregates that may have been precipitated. PBA (5 and 10 mM) treatment however was unable to inhibit the formation of heat and DTT induced larger aggregates (lanes 4 & 5 vs 3), whereas, TUDCA inhibited the formation of larger aggregates in a concentration dependent manner (lanes 6 & 7 vs 3). Consistent with these results, heat and DTT treatment induced maximum aggregation as analyzed by spectrophotometer at 492 nm (Fig. 2B, bar 7). It was efficiently prevented by TUDCA than by PBA (Fig. 2B, bar 10 vs 7–9).

Consistent with the above suggestion that BSA aggregates formed in response to heat and DTT treatment may have been precipitated, it was observed here that BSA protein was found mostly in the precipitate than in the supernatant in heat and DTT treated samples as analyzed by reducing and non reducing gels (Fig. 2C and D, lanes 9 &10). Protein was recovered however in the supernatant fraction when TUDCA was included (lanes 15–18 vs 9 and 10). In contrast, PBA was found to be ineffective in inhibiting BSA precipitation formed in response to heat and DTT treatment and thus the protein remained in the pellet fraction (lanes 11–14 vs 9 and 10). In the presence of SDS-sample buffer, BSA is denatured and disulphide bridges are broken and BSA appears as a monomer at 66 kDa. The differential migration of BSA monomers in control samples (lane 1) in reducing (Fig. 2C) and non-reducing (Fig. 2D) SDS-PAGE may be due to the presence of β-mercaptoethanol, an agent that reduces disulphide bonds and affects the native conformation of the BSA protein. Presence of β- mercaptoethanol slowed down the migration of BSA whereas absence of β-mercaptoethanol promoted faster migration. This is further substantiated by DTT, another reducing agent of protein disulphide groups (lanes 2–6 and 9–18). Overall these findings suggest that TUDCA resists the formation of protein aggregates induced in response to heat, DTT or heat and DTT more efficiently than PBA.

### PBA inhibits but TUDCA enhances trypsin mediated digestion of BSA monomers

**Figure 3:**
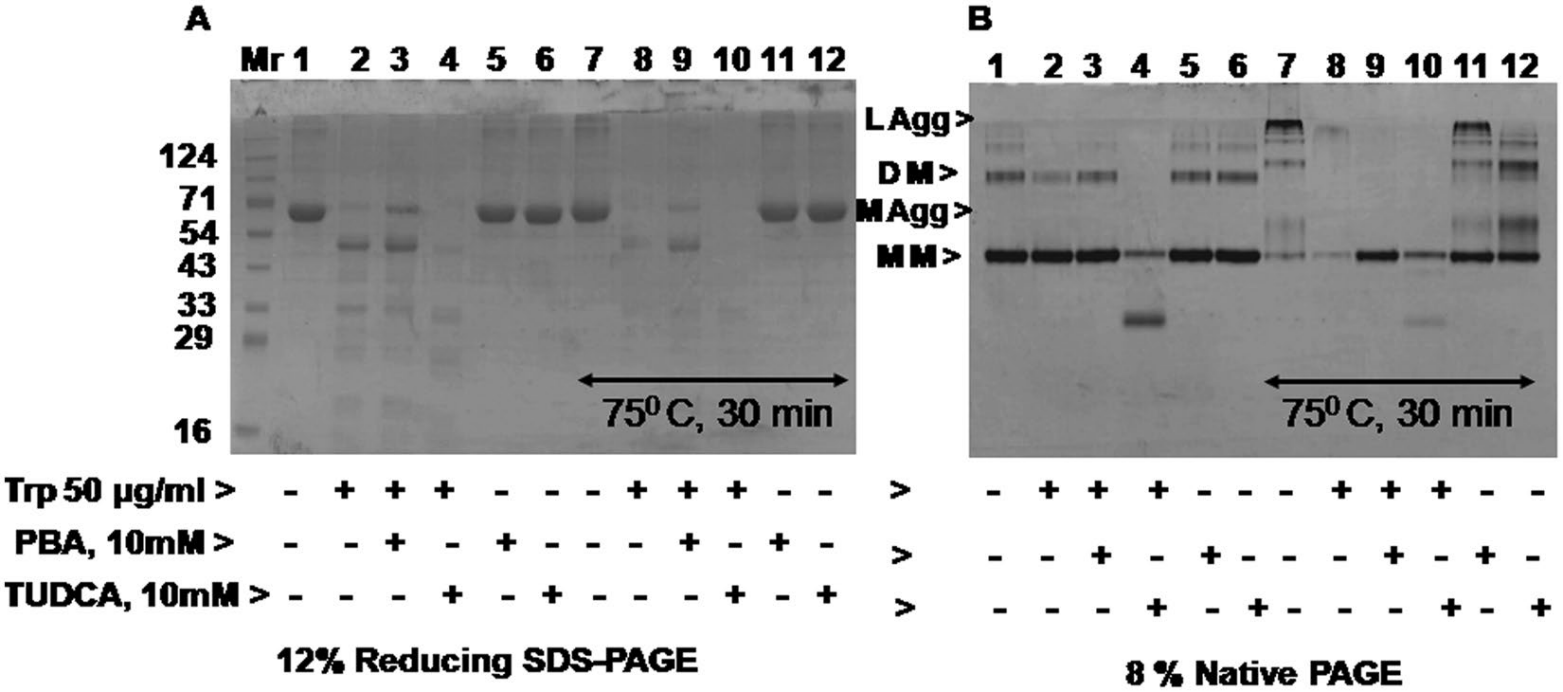
Trypsin mediated BSA digestion in the presence of PBA or TUDCA: Trypsin mediated BSA digestion was analyzed by 12% SDS-PAGE (Panel A) and also by 8% Native-PAGE (Panel B) at 30° C and 75° C for 30 min. BSA (1 mg/ml) was treated with trypsin (50 µg/ml) for 10 min at 30° C in the presence and absence of 10 mM PBA or TUDCA. To determine trypsin activity on aggregated protein, BSA samples were heat treated at 75° C for 30 min in the presence and absence of 10 mM PBA or TUDCA and brought to room temperature before trypsin was added. Digested products were then analyzed by 12% SDS-PAGE and Native-PAGE. Various lanes are as follows: 1, native BSA; lanes 2–6 are native BSA treated with trypsin; trypsin + 10 mM PBA; trypsin + 10 mM TUDCA; 10 mM PBA alone and 10 mM TUDCA alone respectively; lane 7, heat treated BSA at 75° C for 30 min; 8, heat treated BSA + trypsin; 9, BSA + 10 mM PBA + heat + trypsin; 10, BSA + 10 mM TUDCA + heat + trypsin; 11 and 12, BSA + 10 mM PBA + heat and BSA + 10 mM TUDCA + heat respectively.

To determine the protein degradation/digestion, trypsin (50 μg/ml) mediated digestion of native (at 30° C) and aggregated BSA (at 75° C for 30 min) was studied in the presence and absence of PBA and TUDCA. Digested products were analyzed by 12% SDS-PAGE (Fig. 3A) and 8% Native-PAGE (Fig. 3B). During heat treatment, BSA was treated at 75 °C for 30 min, brought to room temperature before trypsin was added. Trypsin digested native BSA into various low molecular wt BSA products (Fig. 3A, lane 2 vs1). Efficiency of BSA digestion by trypsin was somewhat reduced in the presence of PBA (lane 3 vs 2) but it was enhanced by TUDCA (lane 4 vs 2) as determined by the stain intensity of the various bands. However when products of trypsin digested BSA were resolved on native gel (Fig. 3B), it was observed that trypsin digested dimers (DM) of BSA more readily rather than monomers (MM) under those conditions (lane 2 vs1). Presence of TUDCA enhanced trypsin mediated digestion of dimers and also monomers albeit that dimers were digested much more readily than monomers (lane 4 vs 2). In contrast, PBA resisted trypsin mediated digestion of native BSA monomers (lane 3 vs 2). Further, heat treated BSA that contains medium and larger aggregates was digested by trypsin efficiently than native BSA suggesting that aggregated BSA protein may be a better substrate for digestion (lane 8 vs 2). PBA also failed to enhance trypsin mediated digestion of heat treated BSA (lane 9 vs 8). In contrast, the enhanced ability of trypsin to digest BSA in the presence of TUDCA at 30° C was not evident at 75 ° C (lane 10 vs 8). This may be because aggregated proteins are digested by trypsin more efficiently than unfolded or native proteins. Since TUDCA relieves larger aggregates and facilitates formation of smaller aggregates and unfolded proteins, TUDCA’s effect on trypsin mediated digestion of heat treated BSA was not very evident. Further TUDCA appears to promote a structural change in BSA rather than in trypsin protein because pretreatment of trypsin with TUDCA did not enhance any further its ability to digest BSA protein (see Supplementary Fig. S2). Overall these findings suggest that trypsin targets readily the digestion of BSA dimers which is enhanced by TUDCA and inhibited by PBA at 30 °C. In addition aggregated BSA protein appears to be a better substrate for trypsin than native BSA protein.

### PBA but not TUDCA decreases HepG2 cell viability

**Figure 4:**
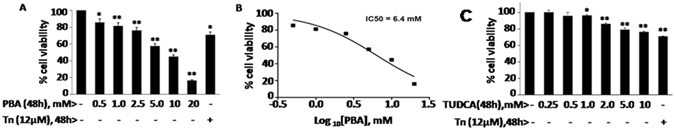
HepG2 cell viability in the presence of PBA or TUDCA: HepG2 cell viability was measured by MTT assay as described in ‘Materials and Methods’. Panel A: Cells were treated with different concentrations of PBA (0.5–20 mM) or with 12 µM tunicamycin for 48 h. Panel B: IC50 value for PBA as determined by using GraphPad Prism 6 software. Panel C: Cell viability in the presence of different concentrations of TUDCA (0.25–10 mM) or 12 µM tunicamycin for 48 h. Data shown are mean ± SD, (n = 3). The asterisks represent statistically significant differences (*P < 0.05, **P < 0.01). P value is calculated by t-test.

HepG2 cell viability, estimated by MTT assays as described in Materials and Methods, decreased with increasing concentrations of PBA and the IC50 value was estimated to be 6.4 mM (Fig. 4A and B). In contrast, the decrease in cell viability in the presence of different concentrations of TUDCA (0.25–10 mM) was found to be approximately 15–20% (Fig. 4C) and thus TUDCA is found less cytotoxic than PBA for HepG2 cells. Tunicamycin, an inhibitor of N-linked glycosylation and that promotes typically endoplasmic reticulum (ER) stress and unfolded protein response (UPR), decreased cell viability approximately 25% at 48 hrs. These findings therefore suggest that PBA, but not TUDCA, is cytotoxic.

### PBA promotes eIF2α phosphorylation, mitigates tunicamycin induced UPR and enhances cell death

**Figure 5:**
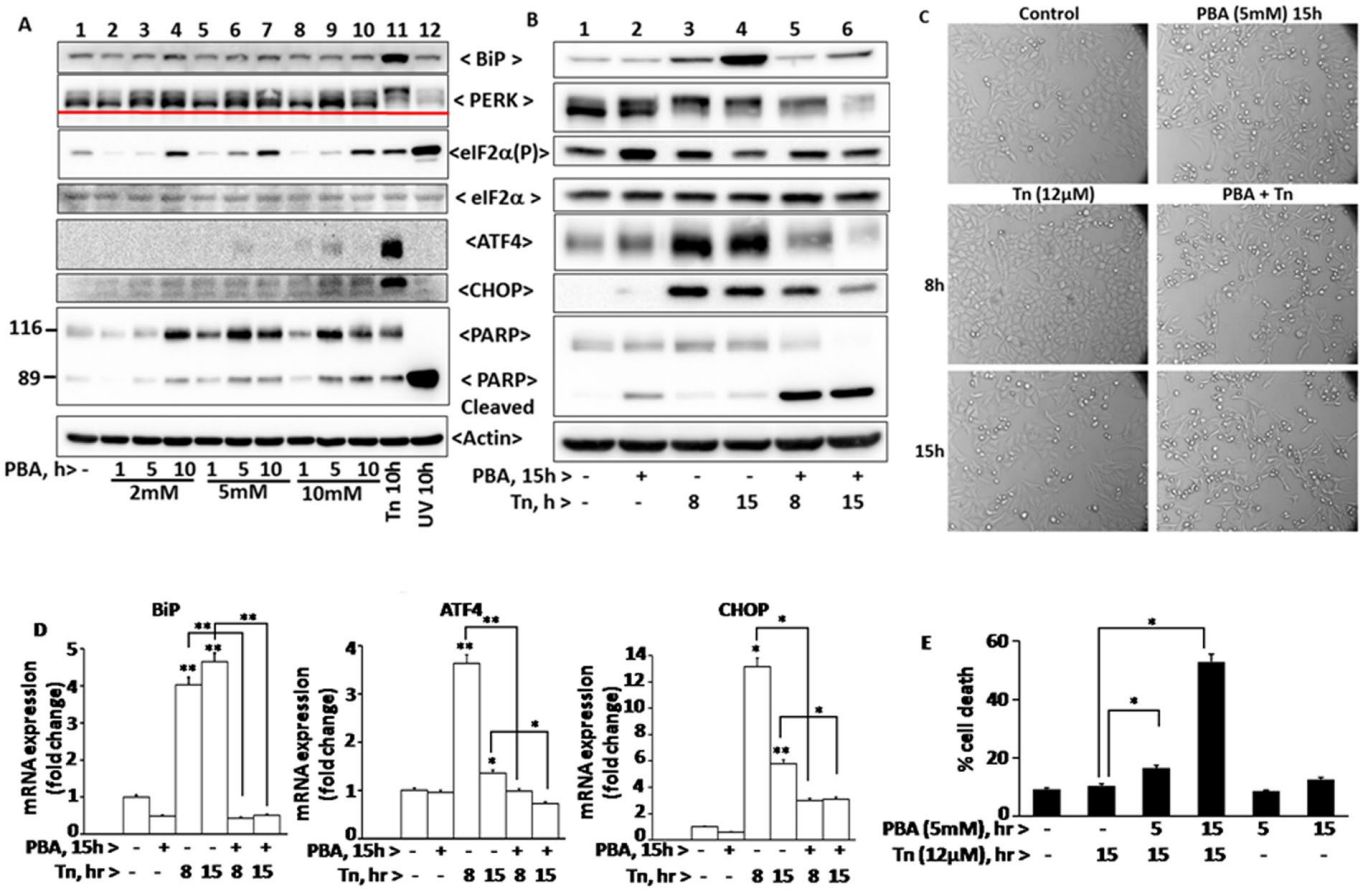
Tunicamycin induced UPR, PARP cleavage, and cell viability in the presence of PBA: In Panel A, HepG2 cells were treated with different concentrations of PBA (2, 5, and 10 mM) for different time periods (1, 5, and 10 h), or with 12 µM tunicamycin, an ER stressor for 10 h, or exposed to UV-B irradiation, a DNA-damaging agent, for 60 seconds and then incubated at 37° C for 10 h. In Panel B, cells were pretreated with 5 mM PBA for 15 h and then treated with 12 µM tunicamycin for 8 and 15 h to determine the effect of PBA on tunicamycin induced UPR. After respective treatments, cells were lysed and proteins were analyzed by their respective antibodies by western blotting. PERK activation or phosphorylation was estimated by its differential mobility on the gel. Phosphorylated PERK migrates slower than unphosphorylated form. Panel C represents analysis of morphological changes observed under inverted microscope at a magnification of 20 x in cells treated with various agents as mentioned in Panel B. Panel D represents quantitative RT- PCR analysis of BiP, ATF4, and CHOP mRNAs in cells for the corresponding treatments as mentioned in panel B. Panel E represents FACS analysis of HepG2 cells which were pretreated with 5 mM PBA for 5 and 15 h, and then incubated with 12 μM tunicamycin for 15 h. Cell death was analyzed by FACS as described in ‘Materials and Methods’. Data shown are mean ± SD, (n = 3). The asterisks represent statistically significant differences (*P < 0.05, **P < 0.01). P value is calculated by t-test.

As a known chemical chaperone, PBA and TUDCA are reported to alleviate UPR, an adaptive signaling pathway evoked by accumulation of unfolded proteins in the ER^37, 46^. However, in our previous studies, we observed that TUDCA prevents stress induced protein aggregation and enhances activation of PERK in a concentration dependent manner^45^. Since the present study also suggests that PBA prevents stress induced protein aggregation like TUDCA, albeit less efficiently, we tested its ability to alleviate tunicamycin induced PERK-eIF2α-ATF4 arm of UPR and cell death (Fig. 5). PBA stimulated however phosphorylation of eIF2α as analyzed by a phosphospecific antibody (Fig. 5A, lanes 1 vs 2–10). 2 mM PBA was sufficient to promote maximum amount of eIF2α phosphorylation within 10 hrs. Since eIF2α phosphorylation is a stress signal and can occur in response to ER and non ER stressors^47^ through the activation of respective eIF2α kinases, we have analyzed here whether PBA induced eIF2α phosphorylation is mediated by PERK activation by comparing PBA effects with tunicamycin and UV-irradiation-treated HepG2 cells. HepG2 cells treated for 10 hrs with 12 μM tunicamycin, a typical ER stressor, stimulated expression of BiP, activation of PERK, eIF2α phosphorylation and expression of transcriptional factors ATF4 and CHOP, the downstream targets of eIF2α phosphorylation (Fig. 5A, lane 11 vs 1) as expected. Time course analysis of tunicamycin induced UPR markers were shown in Supplementary Fig. S3A. In contrast, cells exposed to UV-B irradiation, a DNA damaging agent, for one minute and incubated for 10 hrs, promoted eIF2α phosphorylation but without the expression of BiP, activation of PERK and also expression of ATF4 and CHOP (Fig. 5A, lane 12 vs 11 or 1), as has been previously demonstrated^48^. A time course analysis on the effects of UV-B irradiation induced activation of PERK, eIF2α phosphorylation, expression of BiP, ATF4 and CHOP, and also PARP cleavage were shown in Supplementary Fig. S3B. These results suggest that UV-irradiation, unlike tunicamycin, does not induce UPR markers and turnover eIF2α phosphorylation as it is found increasing with time. PBA did not induce PERK activation or expression of BiP. A very small increase in the expression of ATF4 and CHOP proteins were observed (Fig. 5A). This may be due to preferential translation of basal level of ATF4 transcripts (Fig. 5D, bar diagram 2) in response to eIF2α phosphorylation.

Expression of BiP, ATF4 and CHOP proteins in tunicamycin and PBA treated cells were further correlated to RT-PCR studies (Fig. 5D bar diagram, bars 1–4) suggesting that ATF4 and CHOP expression are regulated by ER stress^42^ and also by eIF2α phosphorylation as suggested^43^. Relative levels of eIF2α phosphorylation that occurs in response to a wide variety of stressors depends on kinase-phosphatase activities. eIF2α phosphorylation was higher in cells treated with PBA for 15 hrs compared to 8 and 15 hrs tunicamycin treated cells (Fig. 5B, lanes 2 vs 1 and 2 vs 3 and 4). This may be due to poor turnover of phosphate on eIF2α in PBA treated cells due to lack of expression of CHOP that in turn stimulates GADD-34, a cofactor of type1 phosphatase. This explanation is also consistent with the higher levels of eIF2α phosphorylation observed in UV-irradiated cells that lack CHOP expression.

Consistent with its ability to decrease cell viability (Fig. 4), PBA treated cells displayed higher levels of cleaved Poly ADP-ribose polymerase (PARP, 89 kDa) in a concentration and time-dependent manner (Fig. 5A, lanes 1 vs 2–10). However PBA or tunicamycin induced PARP cleavage was relatively low when compared to UV-irradiation and is consistent with their ability to promote cell death (Fig. 5A, lane 12 vs 11 or lane 12 vs 2–10). PBA mitigated efficiently tunicamycin induced expression of BiP, ATF4 and CHOP proteins, their respective mRNAs (Fig. 5B, lanes 5 and 6 vs 3 and 4; 5D, bars 5 and 6 vs 3and 4), and also activation of PERK (Fig. 5B) suggesting that it alleviates tunicamycin induced ER stress. However PBA did not mitigate efficiently tunicamycin induced eIF2α phosphorylation. This may be due to a) both PBA and tunicamycin can activate different eIF2α kinases to stimulate eIF2α phosphorylation and b) the low levels of expression of CHOP in PBA + tunicamycin treated cells compared to tunicamycin alone may have reduced the expression of GADD-34 and is reflected accordingly in higher levels of eIF2α phosphorylation. Since PBA mitigated tunicamycin induced UPR markers, we have tested here to determine if it would also mitigate tunicamycin induced PARP cleavage and cell death. But PBA enhanced tunicamycin induced PARP cleavage (Fig. 5B) and it was correlated to cell death as estimated by FACS analysis (Fig. 5E) and also by monitoring cell morphology under inverted microscope (Fig. 5C).

### TUDCA mitigates tunicamycin induced UPR and cell death

**Figure 6:**
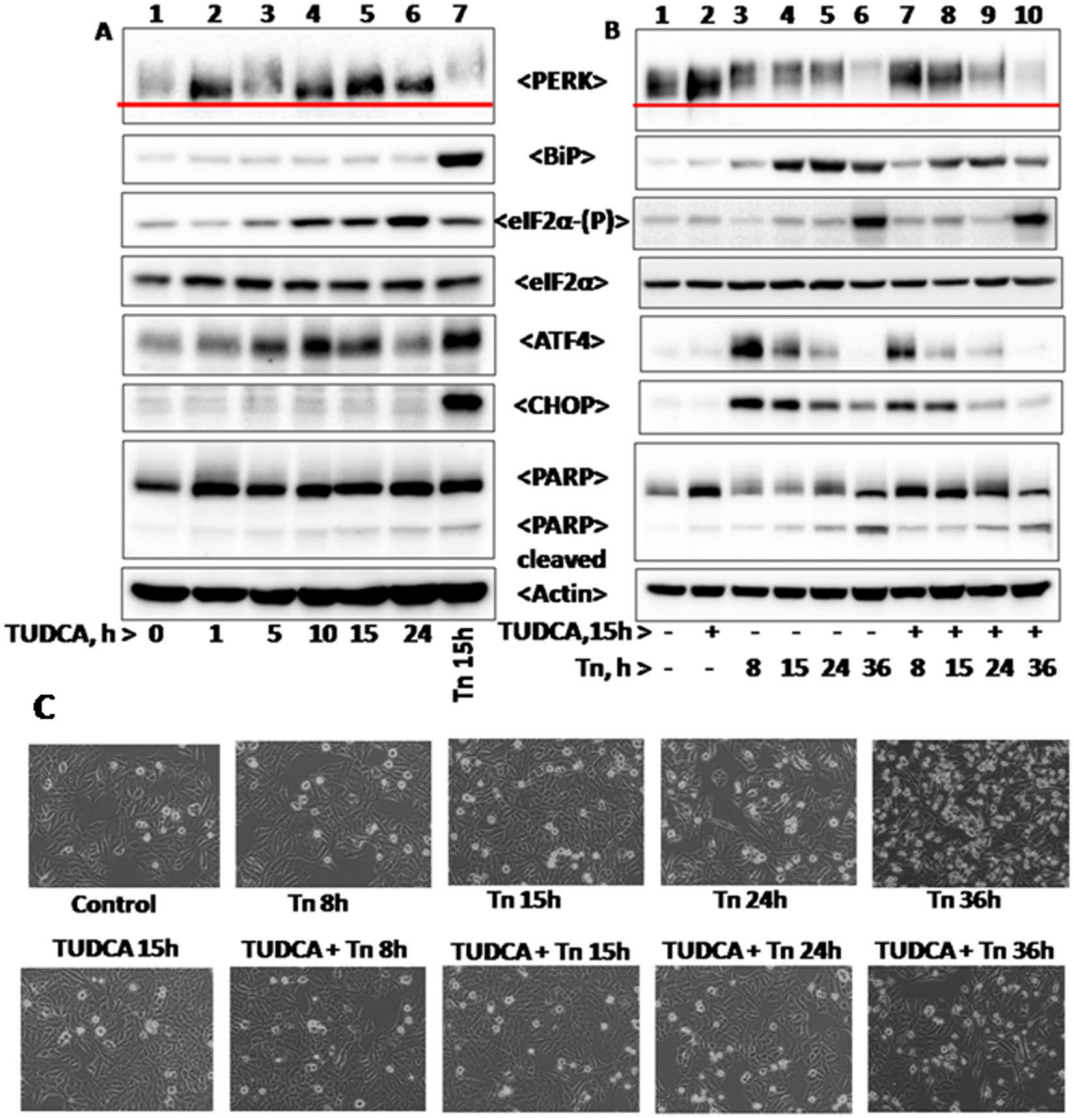
Tunicamycin induced UPR and PARP cleavage in the presence of TUDCA: HepG2 cells were treated with12 µM tunicamycin, 2 mM TUDCA or with TUDCA and tunicamycin for different time periods as shown in the figure and were processed as described in Materials and Methods. Panel A represents time course analysis of different UPR marker proteins in cells treated with 2 mM TUDCA for indicated time periods. Panel B represents UPR status in cells pretreated with 2 mM TUDCA for 15 h and then incubated with 12 µM tunicamycin for different time periods (8, 15, 24, and 36 h) as indicated in the figure. Panel C represents morphology of HepG2 cells in response to corresponding treatments as mentioned in panel B.

Previously we suggested that TUDCA activates PERK in a concentration dependent manner^45^. However we used 2 mM TUDCA here in all our experiments as it was sufficient to overcome tunicamycin induced cell death as analyzed by cell morphology (Fig. 6C). At this concentration, TUDCA promoted mild activation of PERK-eIF2α-ATF4 arm of UPR. However, TUDCA did not induce the expression of BiP or CHOP (Fig. 6A). Consistent with its ability to maintain cell viability, TUDCA did not stimulate PARP cleavage (Fig. 6A). Also the relative level of ATF4 expression induced by TUDCA was higher compared to PBA (Figs 6A vs 5A) and it may be due to its ability to stimulate PERK arm of UPR. TUDCA’s ability to reduce protein aggregation and maintain unfolded and unaggregated proteins may have caused PERK activation. TUDCA’s ability to stimulate PERK activation, eIF2α phosphorylation and ATF4 expression were observed in early time points but not at 24 hrs (Fig. 6A). TUDCA is not a typical ER stressor and it is associated with multiple activities such as maintenance of unfolded proteins, inability to express CHOP and GADD-34 and antioxidant activity. While the first two can result in PERK mediated eIF2α phosphorylation and its increase with time, the antioxidant activity of TUDCA may suppress ATF4 expression with increasing cellular concentration of TUDCA since ATF4 expression is involved in maintaining reducing environment. Alternatively, the relative stability of ATF4 may vary in TUDCA treated cells.

TUDCA also reduced tunicamycin induced PERK-ATF4-CHOP arm of UPR and also the expression of BiP, but not eIF2α phosphorylation (Fig. 6B and see Supplementary Fig. S4 representing relative levels of UPR markers shown in Fig. 6B). The latter may be again due to a reduction in the expression of CHOP that stimulates the expression of GADD-34. Unlike PBA (Fig. 5B), TUDCA protected tunicamycin induced cell death and was consistent with its ability to reduce tunicamycin induced PARP cleavage under those conditions.

### TUDCA inhibits UV-irradiation and PBA induced cell death

**Figure 7:**
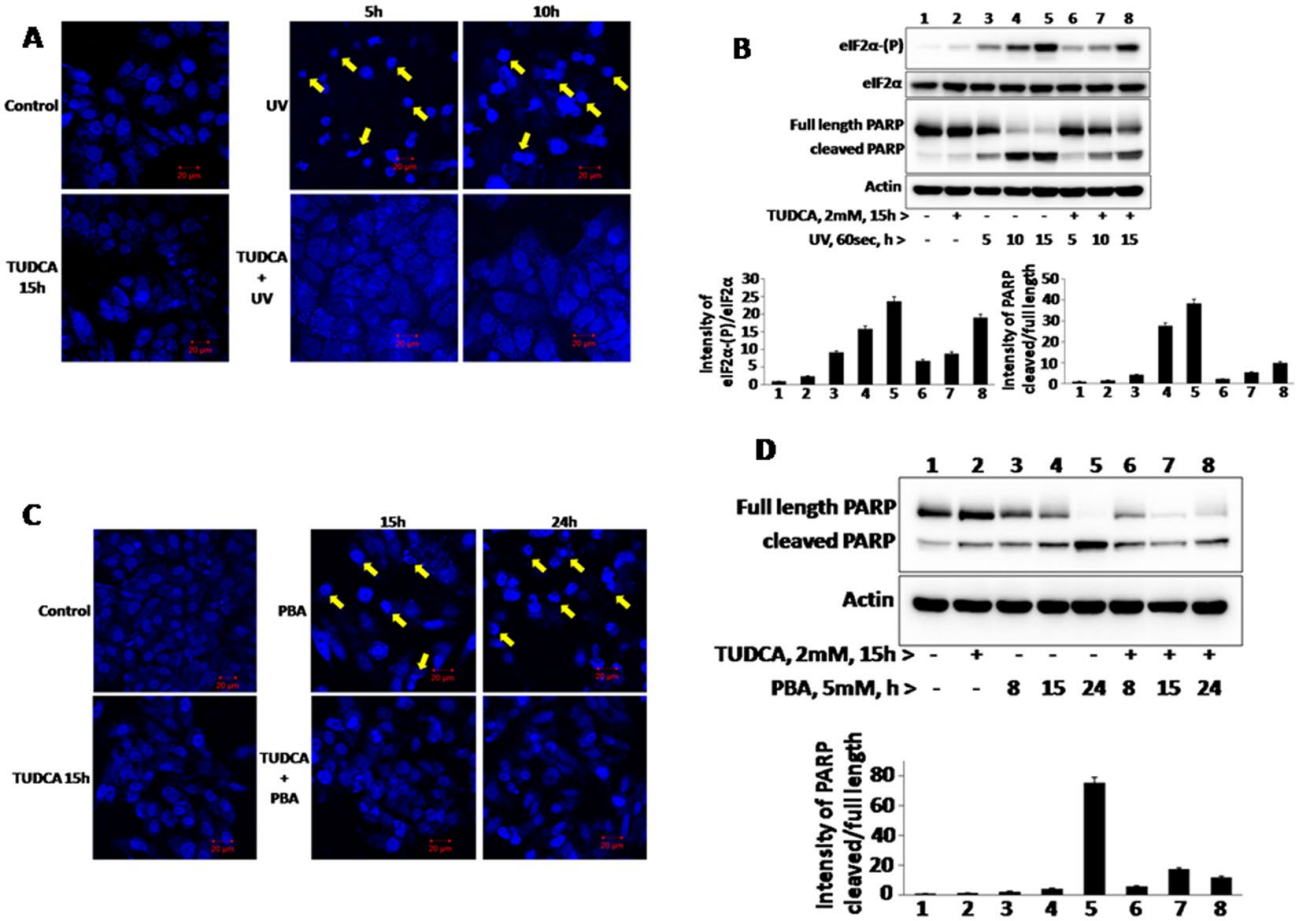
UV-B irradiation or PBA induced nuclear fragmentation and PARP cleavage in the presence of TUDCA: To determine the effect of TUDCA on UV-B irradiation and PBA induced nuclear fragmentation and PARP cleavage, 2 mM TUDCA pretreated cells (15 h) were exposed to UV-B irradiation for 60 seconds (200 J/m^2^) or treated with 5 mM PBA and then cells were incubated at 37° C for 5 and 10 h after UV- irradiation, or 15 and 24 h after PBA treatment. Nuclear fragmentation was analyzed by DAPI staining in confocal microscopy (Panel A and Panel C) as described in ‘Materials and Methods’. Arrow marks indicate fragmented nuclei. PARP cleavage was analyzed for respective treatments by a specific antibody by western blotting (Panel B and D).

**Figure 8:**
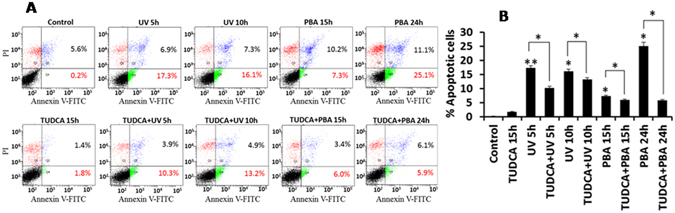
TUDCA inhibits UV-B irradiation and PBA induced apoptosis: TUDCA (2 mM) pretreated HepG2 cells were treated with UV-B irradiation for 60 seconds and incubated at 37° C for 5 and 10 h or with 5 mM PBA for 15 and 24 h. Apoptosis was measured with annexin V-FITC/PI staining by FACS analysis as suggested by manufacturer. Lower right quadrant (Q4) represents early apoptosis, that is, Annexin V-FITC-positive/PI-negative cells; upper right quadrant (Q2) represents necrosis or late-apoptosis, that is, Annexin V-FITC-positive/PI-positive cells. Panel B bar diagram represents per cent of cells undergoing apoptosis as shown in panel A. Data shown are mean ± SD of three independent experiments. *P < 0.05, **P < 0.01. P value is calculated by t-test.

Our results here (Fig. 6) indicating that TUDCA mitigates tunicamycin induced UPR in HepG2 cells is consistent with previous observations that TUDCA mitigates ER stress induced cell death^27, 28^. We also observed here that both UV-irradiation and PBA induced cell death were mitigated by TUDCA as analyzed by nuclear fragmentation (Fig. 7A and C), PARP cleavage (Fig. 7B and D), Annexin V-FITC/PI staining (Fig. 8) and also by monitoring cell morphology under inverted microscope (see Supplementary Fig. S5A and B). These findings therefore suggest that TUDCA mitigates both ER and non-ER stress induced cell death. TUDCA pretreated cells exposed to UV-irradiation displayed reduction in eIF2α phosphorylation when compared to UV-irradiated cells alone in spite of lack of CHOP expression (Fig. 7B). UV-irradiation is shown to stimulate GCN2 kinase^49^ which is activated not only by nutrient deprivation but also by oxidative stress. Hence it is likely TUDCA may be inhibiting the eIF2α kinase activity in UV-irradiated cells through its antioxidant activity. Overall these findings suggest that TUDCA, unlike PBA, is a potent antiapoptotic agent.

## Discussion

In a recent study, we observed TUDCA inhibits stress induced protein aggregation *in vitro* and enhances activation of PERK, the ER-resident eIF2α kinase in HepG2 cells^45^ suggesting that TUDCA apparently stimulates unfolded protein response, a prosurvival signaling pathway that is evoked in response to the accumulation of unfolded proteins in ER. However TUDCA is known for its ability to mitigate ER stress rather than to enhance ER stress^37, 46, 50^. This has prompted us to evaluate TUDCA along with PBA, another chemical chaperone, for their abilities to mitigate stress induced protein aggregation, ER stress induced UPR and analyze their overall effects on cell survival and death in the presence of ER and non-ER stressors like tunicamycin and UV-irradiation respectively^47^. Our studies here reveal that both chemical chaperones reduce heat induced protein aggregation (Fig. 1). However, PBA, unlike TUDCA, cannot reduce the heat and DTT induced aggregation of BSA (Fig. 2) suggesting that TUDCA appears to be more effective compared to PBA in preventing stress induced aggregation of BSA protein *in vitro*. This interpretation is also consistent with the ability of TUDCA to reduce significantly the large BSA aggregates formed in response to heat than by PBA (Fig. 1A). TUDCA, but not PBA is also observed here to enhance trypsin mediated BSA digestion *in vitro* (Fig. 3) and is consistent with the fact that bile salts aid protein digestion^51^.

Since chemical chaperones are known to mitigate ER stress in physiological conditions^29^ and indirectly promote protein folding, we analyzed here the ability of TUDCA and PBA independently on UPR activation and also for their abilities to mitigate tunicamycin induced PERK-eIF2α-ATF4 arm of UPR and cell death. Being a typical ER stressor, tunicamycin treatment resulted in the activation of PERK (as estimated by its decreased mobility on SDS-PAGE), eIF2α phosphorylation and ATF4 expression (Fig. 5). In addition, it also induces the expression of ER chaperone, BiP and CHOP, a transcriptional factor which is also regulated by ER stress and eIF2α phosphorylation^17^. When TUDCA and PBA treated cells were analyzed for various UPR markers, both of them stimulated eIF2α phosphorylation and ATF4 expression without inducing BiP and CHOP (Figs 5 and 6) suggesting that they cannot evoke typical UPR like tunicamycin. Further analysis revealed that TUDCA, but not PBA, induced eIF2α phosphorylation is mediated by mild PERK activation. This is consistent with our findings here that TUDCA can prevent stress induced aggregation of proteins much more efficiently than PBA and maintain unfolded proteins that can activate PERK. TUDCA but not PBA’s ability to promote mild PERK activation is also consistent with the expression levels of ATF4 which is much higher in TUDCA treated cells than in PBA treated cells (Figs 5 and 6). ATF4 expression, that in turn can stimulate genes involved in redox metabolism and autophagy, may also be regulated by the antioxidant activity of TUDCA. Reduction in ATF4 in cells treated with TUDCA for longer duration (Fig. 6A) may be due to the antioxidant activity associated with TUDCA which can increase thiol containing proteins, scavenge hydroxyl radicals and can also activate metallothionein promoters in hepatocytes and in HepG2 cells^15, 52^. Enhanced levels of eIF2α phosphorylation observed in  cells treated with TUDCA and in UV-irradiation compared to tunicamycin may be a consequence of little or no expression of CHOP that in turn targets the expression of GADD-34, a cofactor of eIF2α phosphatase (Figs 5 and 6).

Although the mechanistic details are not clear, both chemical chaperones however are found to mitigate the expression of BiP, ATF4 and CHOP induced by tunicamycin, an ER stressor (Figs 5 and 6) as suggested earlier^37^. However, PBA unlike TUDCA, promotes PARP cleavage and cell death (Fig. 5). This may be due to the inhibition of histone deacetylase activity associated with PBA^53^. Further, our studies here point out that TUDCA’s antiapoptotic activity may not be limited to ER stress induced cell death as it can protect cell death mediated by UV-irradiation, a DNA damaging agent, and PBA that inhibits histone deacetyl transferase activity (Fig. 7). It is likely that TUDCA’s antiapoptotic activity may be because of its ability to mitigate ER stress induced UPR as in tunicamycin treated cells, or mitigate ROS generation as in UV-irradiated cells because of its antioxidant activity^54^. TUDCA’s antiapoptotic ability to resist even UV-irradiation induced cell death in transformed HepG2 cells suggest that it promotes cancer cell dormancy thus raising concern about its therapeutic potential to treat diseases other than biliary cirrhosis as has been suggested^55^. In contrast, PBA’s ability to stimulate HepG2 cell death and reduce UPR may play an important role in limiting tumor cell progression^56, 57^.

## Materials and Methods

### Chemicals and antibodies

BSA (Bovine Serum Albumin) from Himedia (India), PBA and trypsin from Sigma, TUDCA and tunicamycin from Calbiochem (India) were used throughout this study except as otherwise mentioned. Rabbit polyclonal anti- BiP antibody (cat no# Sc-13698) and anti-eIF2α antibody (cat no# Sc-11386) were purchased from Santa Cruz Biotechnology, Inc, USA. Rabbit monoclonal phospho-eIF2α (ser-51) antibody (cat no# NB110-56949) was obtained from Novus Biologicals, USA. Rabbit monoclonal anti-PERK antibody (cat no# 5683S), rabbit monoclonal anti-ATF4 antibody (cat no# 11815S), mouse monoclonal anti-CHOP antibody (cat no# 2895S) and rabbit monoclonal anti-PARP antibody (cat no# 9532S) were bought from cell signaling technologies, USA. Mouse monoclonal anti-actin antibody (cat no# A5441-100UL) was obtained from Sigma.

### Cell culture treatments and cell extracts preparation

HepG2 cells were grown at 37° C with 5% CO_2_ in DMEM medium supplemented with 10% fetal bovine serum, 50U of penicillin G, and 50 µg/ml streptomycin. Cells were seeded in 60 mm cell culture dishes a day before treating them with different concentrations of PBA, TUDCA or tunicamycin for different time periods as indicated in the legends to figures. Cells were also treated with UV-B irradiation for 60 sec corresponding to 200 Joules/m^2^. After treatments, cells were harvested and washed with 1x PBS (phosphate buffered saline) and lysed in lysis buffer containing 20 mM Tris-HCl, 80 mM KCl, 1 mM EDTA, 1 mM EGTA, 0.5% NP-40, 1 mM DTT and protease inhibitors (10 µg/ml pepstatin, 10 µg/ml aprotinin, 250 mM PMSF). Cells were incubated on ice for 10 min, vortexed for 15 min at 4° C, and centrifuged at 12,000 rpm for 20 min. Supernatant was collected and used for western blot analysis or stored at −70° C.

### Cell viability assay

HepG2 cell viability was measured by MTT(3-(4, 5-dimethylthiazol-2-yl)-2, 5-diphenyltetrazolium bromide)^58^ assay. Briefly, cells were seeded in 96 well plate in 100 µL of DMEM medium at a density of 1x 10^4^/well one day before the experiment. Confluent cells were treated with different concentrations of PBA or TUDCA different time points as mentioned in the legends to figures. After treatment, cells were washed thrice with PBS and then incubated with 10 µL of 5 mg/ml MTT solution in PBS for 4 h at 37°C in 5% CO2 incubator. Viable cells metabolize the yellow MTT into the insoluble formazan crystals. Supernatants were discarded and 200 µL of DMSO was added to each well to dissolve formazan crystals. Plates were shaken for 10 min in dark and absorbance was measured at 570 nm using a microplate reader. All experiments were done in triplicates for at least three times.

### FACS analysis

A) PI staining: To determine live and dead cells in response to different reagents, cells were stained with propidium iodide and analyzed by FACS. Cells were treated with different reagents, harvested at 1,200 rpm for 8 min at 4°C, washed with PBS and fixed with 500 μl of 70% ethanol for overnight at 4°C. Cells were then washed with PBS, resuspended in PBS containing 50 μg/ml propidium iodide, 1% triton X-100 and 50 μg/ml RNase A and incubated in dark for 1 h at 37°C. After incubation, cells were centrifuged at 1,200 rpm for 8 min and washed again with PBS, resuspended in sheath fluid and analyzed using BD Biosciences flow cytometer.

B) Annexin V –FITC/PI staining: FACS analysis was also performed to distinguish between live and apoptotic cells by using annexin V-FITC and PI staining (Invitrogen Ltd) as per manufacturer’s instructions. FITC- labelled annexin V binds to membrane phosphatidylserine and propidium iodide binds to cellular DNA. After treatments, cells were washed with PBS and resuspended in 100 μL of 1x annexin binding buffer. Later 5 μL of annexin V and 1 μL of PI was added and incubated for 15 min at room temperature. After incubation, 400 μL 1× binding buffer was added and measured the fluorescence of annexin V-FITC and PI in BD LSRFORTESA flowcytometer. 10,000 events from each sample were acquired to ensure adequate data.

### Real-Time PCR analysis

Total RNA was isolated from HepG2 cells using TRIZOL reagent (Ambion RNA by life Technologies) according to manufacturer’s instructions. Reverse transcription was performed using verso cDNA synthesis Kit (Thermo Scientific, Cat no#AB-1453/A). The expression levels of human BiP, ATF4, CHOP, and 18 s rRNAs were determined using specific primers as indicated in Supplementary Table S1. The USB VeriQuest SYBR Green qPCR Master Mix was used to detect the quantitative real-time PCR products according to the manufacturer’s instructions. Real-time PCR was performed on Applied Biosystems 7300 Real-Time PCR System. The incubation conditions were as follows: pre-denature at 95° C for 10 min, followed by 4° Cycles of 15 sec at 95° C, annealing for 60 sec at 60 °C, and extension for 60 sec at 60 °C. PCR was performed for each sample in triplicate for the target genes and 18 s rRNA.

### ANS fluorescence

180 µM of 1- aniline-naphthalene- 8- sulfonate (ANS) was added to BSA (0.2 mg/ml) in the presence and absence of different concentrations of PBA, TUDCA, and DTT as mentioned in the legends to figures. Samples were incubated at room temperature for 30 min and then fluorescence emission spectra was collected between 400 to 650 nm in spectrofluorimeter (Fluoromax-3) by exciting ANS at 365 nm at 25° C.

### Trypsin mediated BSA digestion

Trypsin (50 µg/ml) was added to BSA (1 mg/ml) or BSA treated PBA or TUDCA for 30 min, incubated for 10 min at 30° C, immediately remove the samples and stopped the reaction by addition of SDS or Native PAGE loading dye. To determine trypsin action on aggregated protein samples, BSA was heat treated at 75° C for 30 min, sample was then brought to room temperature and then incubated with trypsin for 10 min at 30° C. Samples were analyzed by 8% Native or 12% SDS-PAGE. Gels were stained with coomassie brilliant blue R250.

### Protein aggregation assay or turbidity assay

0.2% BSA (w/v) was prepared in phosphate buffer saline (PBS) at pH 7.4. Samples were incubated at 75° C to induce protein unfolding and subsequent aggregation in the presence and absence of 5 and 10 mM PBA, 10 mM TUDCA, 5 mM DTT, or DTT and TUDCA or PBA. BSA turbidity caused by heat or heat and DTT was measured at 492 nm in a Shimadzu UV-visible spectrophotometer.

### DAPI staining

Cells (1 × 10^5^) were plated onto cover slips in 35 mm cell culture dishes and cultured with complete medium. After treatments, cells were fixed with 4% paraformaldehyde for 20 min at room temperature. Cells were washed with PBS and permeabilized with 0.2% triton X-100 in PBS for another 20 min at 4° C. Cells were washed again with PBS and stained with DAPI (4′,6-Diamidino-2-Phenylindole) (2 μg/mL). Nuclear fragmentation of cells was examined by confocal microscope (Zeiss). Apoptotic cells were identified as having condensed and fragmented nuclei. All images were captured at by using 40x magnification lens.

### Gel Electrophoresis

Heat or heat and DTT induced BSA aggregation, and trypsin digestion was analyzed by using Native-PAGE without SDS and β-mercaptoethanol, or by non-reducing SDS-PAGE without β-mercaptoethanol or by regular SDS-PAGE^59^. Electrophoresis was performed at 80 volts in Native-PAGE running buffer without SDS, Gels were stained with coomassie brilliant blueR solution.

### Western blotting

Cells were treated with different reagents for different time periods, harvested and cell extracts were prepared. Cell extract proteins were separated on SDS-PAGE and transferred to nitrocellulose membrane and probed by respective primary antibodies and horse radish peroxidase conjugated secondary antibodies. Chemiluminescence procedure was used for detection according to the manufacturer’s recommendations.

### Statistical analysis

All experiments were performed at least three times, and representative results are shown. All data were shown as the mean ± SD (standard deviation) of three samples (n = 3). Significance was calculated using student’s t-test.

## Electronic supplementary material

Supplementary Information
