# Supplementary material for: Chemical chaperone, TUDCA unlike PBA, mitigates protein aggregation efficiently and resists ER and non-ER stress induced HepG2 cell death
Source: Sci Rep. 2017 Jun 19;7:3831. doi: 10.1038/s41598-017-03940-1 (PMC5476595; doi:10.1038/s41598-017-03940-1)
Supplement: Supplementary file 1 — Supplementary Information [file 41598_2017_3940_MOESM1_ESM.doc]

**Ref: SREP-17-00089 Revised 30 March, 2017**

**Supplementary Information**

**Chemical chaperone, TUDCA unlike PBA, mitigates protein aggregation efficiently and resists ER and non-ER stress induced HepG2 cell death**

Jagadeesh Kumar Uppala, Amina R. Gani, Kolluru V.A. Ramaiah*

**Supplementary Fig. S1**

**
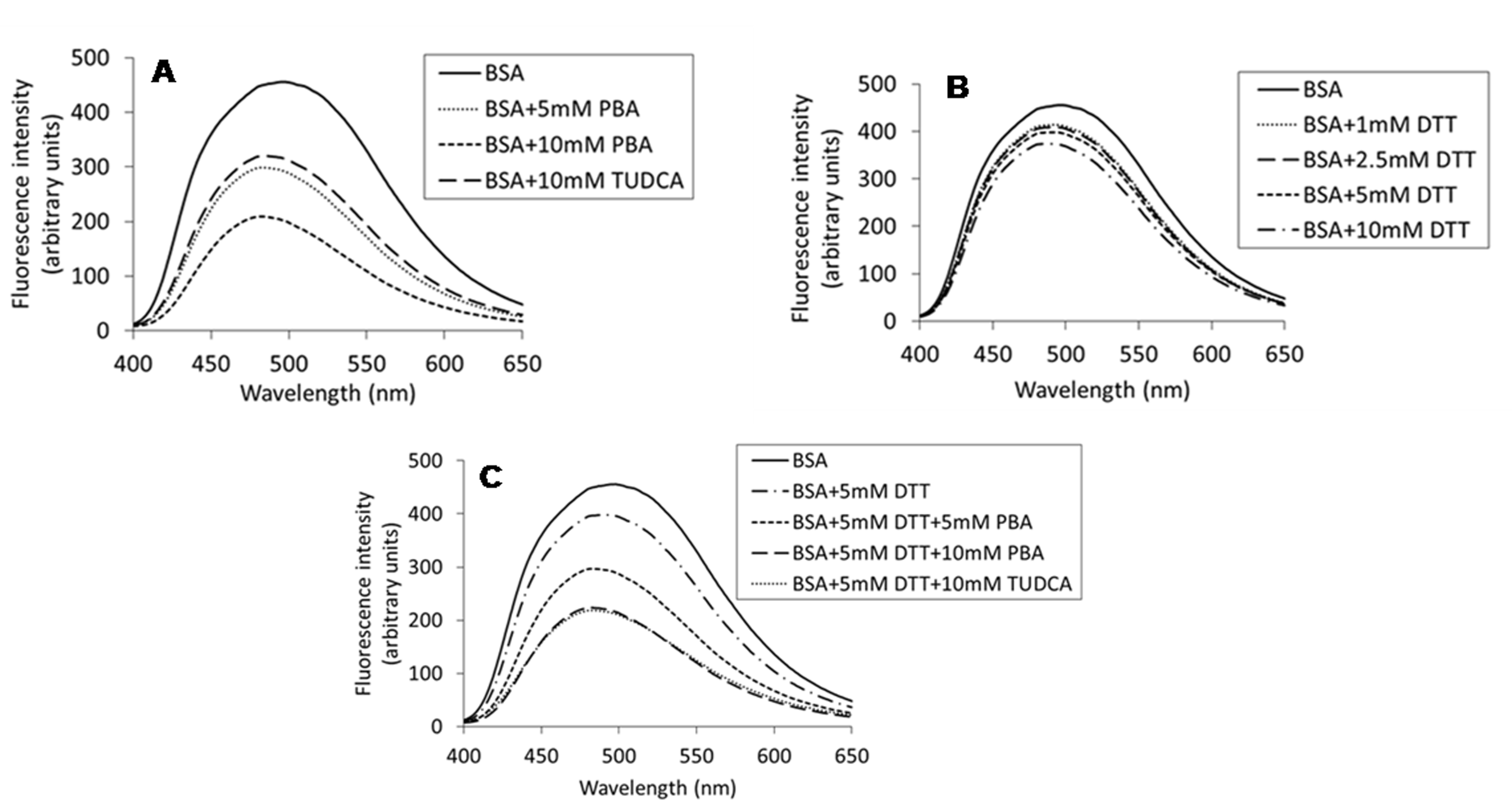
**

**Supplementary Fig. S1: ANS fluorescence emission spectra of BSA in the presence of PBA, TUDCA and DTT alone and in the presence of DTT and PBA or DTT and TUDCA**: ANS fluorescence emission spectra of BSA was measured from 400 to 650 nm at λ_ex_ = 365 nm in the presence and absence of PBA (5 mM and 10 mM) or 10 mM TUDCA (**Panel A**), in the presence of different concentrations of DTT (1, 2.5, 5, and 10 mM) (**Panel B**), and in the presence of DTT and PBA or TUDCA (**Panel C**)

**Supplementary Fig. S2**

**
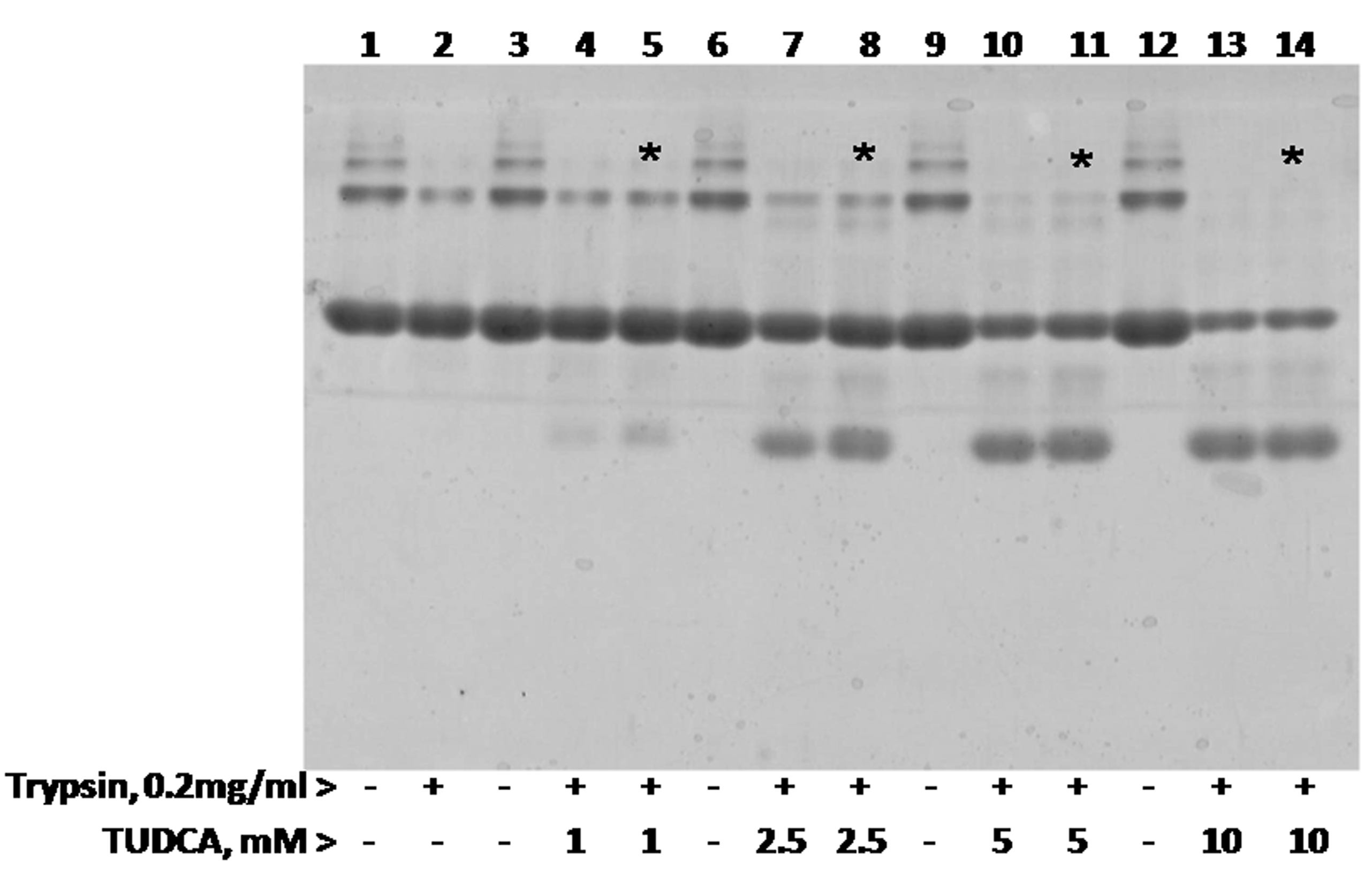
**

**Supplementary Fig. S2:** **BSA digestion by TUDCA pretreated trypsin:** To determine the difference in BSA digestion by trypsin, 1 mg/ml BSA was pretreated with different concentrations of TUDCA and then trypsin (0.2 mg/ml) was added (lanes without asterisk) or TUDCA pretreated trypsin was added to BSA (lanes with asterisk). 1h pretreatment was carried out at room temperature and is followed by 15 min digestion by trypsin. Digested products were analyzed on 12% Native-PAGE Various lanes are as follows: Lane 1, native BSA; 2, BSA+ trypsin; 3, native BSA; 4, 1 mM TUDCA pretreated BSA + trypsin; 5, 1 mM TUDCA pretreated trypsin + BSA; 6, native BSA; 7, 2.5 mM TUDCA pretreated BSA+ trypsin; 8, 2.5 mM TUDCA pretreated trypsin + BSA; 9, native BSA; 10, 5 mM TUDCA pretreated BSA+ trypsin; 11, 5 mM TUDCA pretreated trypsin + BSA; 12, native BSA; 13, 10 mM TUDCA pretreated BSA+ trypsin; 14, 10 mM TUDCA pretreated with trypsin + BSA. BSA digestion by TUDCA pretreated trypsin was shown by asterisk (*).

**Supplementary Fig. S3**

**
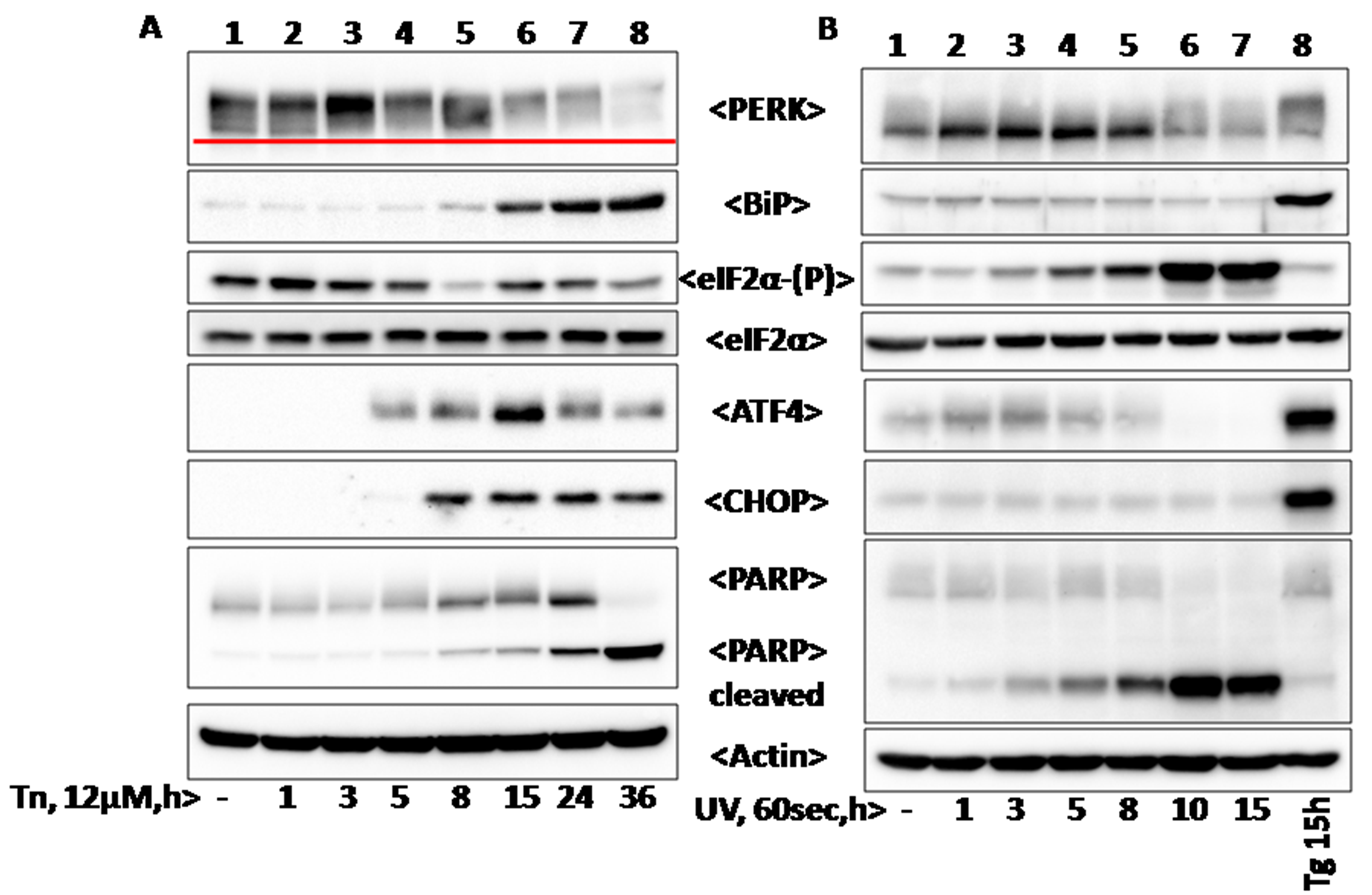
**

**Supplementary Fig. S3: UPR and PARP cleavage mediated by tunicamycin and UV-irradiation:** UPR markers that include activation of PERK, phosphorylation of eIF2α and expression of BiP, ATF4, and CHOP are analyzed at different time points (1, 3, 5. 8, 15, 24 and 36h) in HepG2 cells which are treated with 12 μM tunicamycin or 200 J/ m^2^ UV-B irradiation or 1 μM thapsigargin. Cell extracts were also examined for PARP cleavage, a measure for cell death by using an anti-PARP antibody. UV-irradiated cells displayed eIF2α phosphorylation without PERK activation or expression of BiP, ATF4 and CHOP. PARP cleavage was observed in tunicamycin and UV-irradiated cells.

**Supplementary Fig. S4**

**
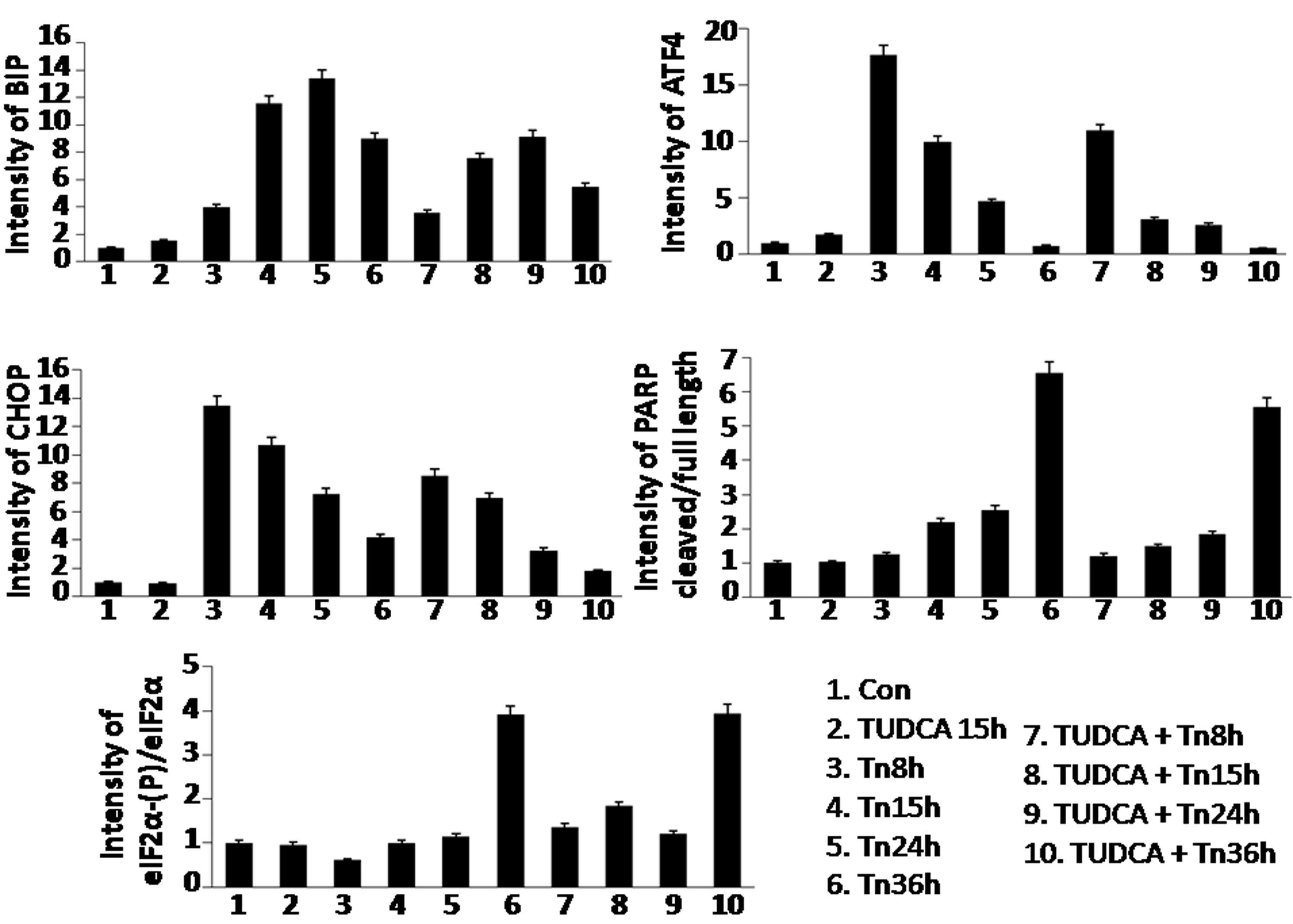
**

**Supplementary Fig. S4: Quantification of UPR markers in the presence of TUDCA and tunicamycin.** The relative levels of eIF2α phosphorylation, expression of BiP, ATF4, CHOP and cleaved PARP observed in TUDCA and tunicamycin treated cells as shown **in Fig. 6B** were quantified by using Image J software and plotted in the form of bar diagrams.

**Supplementary Fig. S5**

**
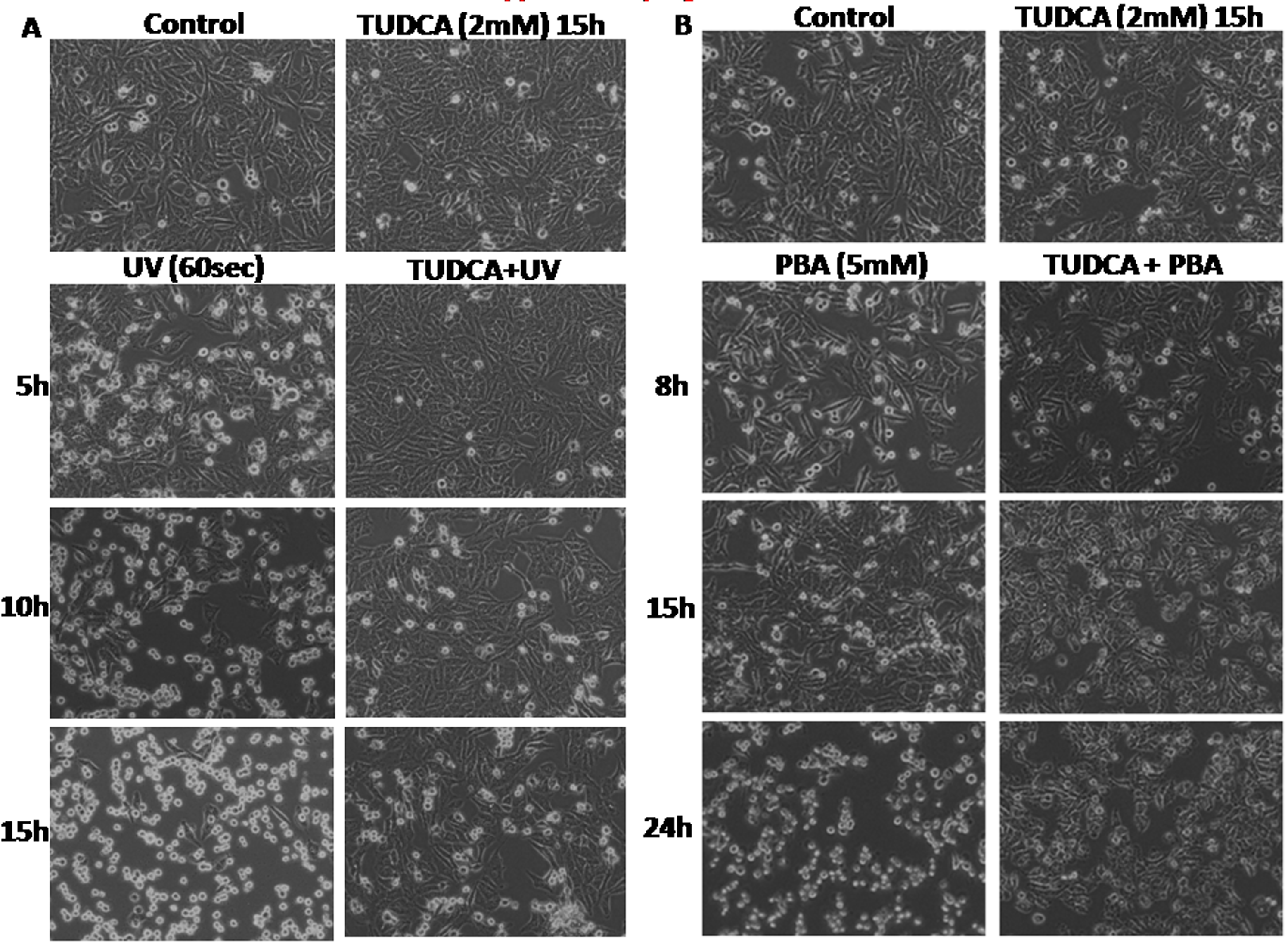
**

**Supplementary Fig. S5: Morphology of TUDCA pretreated HepG2 cells exposed to UV-B irradiation or subjected to PBA treatment.**

Morphology of the HepG2 cells treated with UV-B irradiation (200 J/ m^2^ or 60 seconds) or with 2 mM TUDCA or with 5 mM PBA for different time periods, as indicated in Fig. 7B and D in the main text, were analyzed by inverted microscope and the results here correspond to the PARP cleavage shown in the above Figs.

**Supplementary Table. S1**

| S.No | Gene | Primer Sequence |
| --- | --- | --- |
| 1 | CHOP FP | CCT GGA AAT GAA GAG GAA GAA TC |
|  | CHOP RP | ACT GGA ATC TGG AGA GTG AGG |
| 2 | ATF4 EXP FP | TCC AAC AAC AGC AAG GAG GAT G |
|  | ATF4 EXP RP | TCC AAC GTG GTC AGA AGG TCA TC |
| 3 | BiP FP | ACC ACC TAC TCC TGC GTC |
|  | BiP RP | TTG GAG GTG AGC TGG TTC T |
| 4 | 18s rRNA FP | GAG CGA AAG CAT TTG CCA AG |
|  | 18s rRNA RP | GGC ATC GTT TAT GGT CGG AA |
